# The possible role of the vasopressin system in hematopoiesis

**DOI:** 10.1038/s41598-024-55772-5

**Published:** 2024-03-01

**Authors:** Fredrika Schill, Gunnar Engström, Olle Melander, Simon Timpka, Sofia Enhörning

**Affiliations:** 1 Department of Cardiology, Skåne University Hospital, Carl-Bertil Laurells Gata 9, 214 28 Malmö, Sweden; 2 Department of Clinical Sciences, Lund University, Jan Waldenströms Gata 35, 214 28 Malmö, Sweden; 3 Department of Internal Medicine, Skåne University Hospital, Jan Waldenströms Gata 11 A, 214 28 Malmö, Sweden; 4 Perinatal and Cardiovascular Epidemiology, Department of Clinical Sciences Malmö, Lund University, Jan Waldenströms gata 35, 214 28 Malmö, Sweden; 5 Department of Obstetrics and Gynecology, Skåne University Hospital, Jan Waldenströms gata 47, 214 28 Malmö, Sweden

**Keywords:** Biomarkers, Biomarkers, Endocrinology, Medical research, Risk factors

## Abstract

Vasopressin is a pleiotropic hormone that controls body fluid homeostasis. Vasopressin has also been proposed to be involved in erythropoiesis, thrombocyte activity and inflammation. However, whether increasing vasopressin is associated with changes in hematopoietic markers is not known. To evaluate this gap of knowledge we measured the vasopressin marker copeptin and markers of erythropoiesis (erythrocyte count, hemoglobin (Hb), red blood cell distribution width (RDW), mean corpuscular volume (MCV), erythrocyte volume fraction (EVF)), leukocyte count (total count, lymphocytes, neutrophils) and thrombocyte count in 5312 participants from the Swedish CArdioPulmonary bioImage Study (SCAPIS). The associations between increasing copeptin tertile and the hematopoietic markers were analyzed in multivariate linear regression analyses. We found that increasing copeptin tertile was significantly (*p* < 0.001) associated with increasing erythrocytes, RDW, EVF, Hb, leukocytes and neutrophils after adjustment for age, sex, current smoking, prevalent diabetes, hypertension, creatinine, body mass index and physical activity. Increasing copeptin tertile was, however, not associated with change in MCV, lymphocyte or thrombocyte count. In conclusion, we found that increasing copeptin levels are positively associated with markers of erythropoiesis and leukocyte count in the general population. These results warrant further research on possible mechanistic effects of vasopressin on hematopoiesis.

## Introduction

The pleiotropic hormone vasopressin is known to regulate body fluid homeostasis tightly and is essential in the physiological response to loss of body fluid such as dehydration or hemorrhage. Vasopressin is synthesized by magnocellular neurosecretory cells in the supraoptic and paraventricular nuclei of the hypothalamus and is further released from the posterior pituitary gland after stimuli and activation of neurons located in the organum vasculosum of the lamina terminalis and the subfornical organ^1^. Vasopressin secretion is stimulated by osmotic or hypovolemic stimuli and succeeded by vasopressin induced water conservation in the kidneys and vasoconstriction through effects on vascular smooth muscle cells. Vasopressin also stimulates hemostasis through activation of several coagulation factors and by mediating thrombocyte aggregation^2,3^. Vasopressin acts through three receptors (AVPR1A, AVPR1B, AVPR2) found on a wide range of cell types, including renal epithelial cells, vascular endothelial cells, hepatocytes, myocytes and several hematopoietic cells. Receptor stimuli exerts numerous effects, some of which are still poorly understood. The fact that vasopressin seems to counteract blood loss by several mechanisms has led to the question of whether vasopressin may be involved in the generation of red blood cells. In one study, Mayer et al. found that individuals lacking vasopressin, i.e. suffering from central diabetes insipidus, were prone to anemia^4^. This was, however, contradicted by a similar study by Winzeler et al^5^. Apart from potential effects on thrombocytes and erythrocytes, vasopressin is also suggested to be of importance in inflammation through effects on leukocytes. Vasopressin receptors are found on human leukocytes where they have been found to mediate both pro- and anti-inflammatory effects^6,7^. Even though there are proposed links between the vasopressin system and several hematopoietic cell types, these links are not well understood. For example, no previous study examines whether vasopressin is associated with altered hematopoietic markers in the general population.

The commonly used method of measuring vasopressin is to analyze the C-terminal part of the vasopressin precursor peptide copeptin. Copeptin has been shown to correspond well with vasopressin levels and can be reliably measured in plasma^8^. In this study, we aim to map the associations between copeptin and several markers of erythropoiesis: erythrocytes, hemoglobin (Hb), erythrocyte volume fraction (EVF), red blood cell distribution width (RDW), mean corpuscular volume (MCV)), leukocytes, lymphocytes, neutrophils and thrombocytes.

## Methods

### Ethics declaration

In this cross-sectional cohort study we analyzed complete case data from participants of the Swedish cardiopulmonary bioimage study (SCAPIS). The study was performed in line with ethical standards laid down in the 1964 Declaration of Helsinki and its later amendments and other relevant guidelines. All of the study participants have given an informed and written consent. The study was approved by the Swedish Ethical Review Authority (# 2018-979).

### Study population description

SCAPIS is a national collaboration between six Swedish universities where the cardiopulmonary health of a large cohort of middle-aged men and women was evaluated. A random selection of 30,154 individuals aged 50–64 residing in the six respective cities (Gothenburg, Linköping, Malmö, Stockholm, Umeå and Uppsala) were recruited between 2013 and 2018. The overall participation rate was 50%. The participants were screened for cardiopulmonary risk factors including anthropometrics, lifestyle questionnaires and fasting blood sampling. Several types of imaging were also performed. Each regional site could include optional examinations in addition to the core protocol. The present study uses data from the SCAPIS Malmö cohort (total number = 6251), in which both copeptin and hematopoietic markers were analyzed.

### Data collection and definitions of co-variables

The age of participants was collected at recruitment and analyzed as age in years. Participants’ weight and height were registered by study staff on site. Weight was measured with participants in light clothing, using calibrated scales, and body mass index (BMI) was calculated as kg/m^2^. Blood pressure was measured in the brachial artery of both arms after five minutes of supine rest and calculated as the average of two stable measurements (difference < 10 mmHg) of the arm with the highest value. Hypertension was defined as having a brachial systolic blood pressure of ≥ 140 mmHg, diastolic blood pressure of ≥ 90 or use of antihypertensive medication. Data on smoking status (yes or no) and leisure time physical activity the last 12 months was collected through self-administered questionnaires. In the questionnaire the participants were asked how much they moved and exerted themselves physically during their leisure time. The answers were graded in a four-grade scale ranging from self-reported sedentary lifestyle to regular intense physical activity according to the Saltin-Grimby Physical Activity Level Scale^9^. Prevalent diabetes mellitus was defined as fasting plasma glucose ≥ 7.0 mmol/L, HbA1C ≥ 48 mmol/mol or use of antidiabetic medication. Anemia was classified as having a Hb concentration of less than 130 g (g)/liter (L) for men and 120 g/L for women according to the World Health Organisation’s definition.

### Laboratory measurements

A venous blood sample was collected from participants after an overnight fast and was used for the analysis of high-sensitive C-reactive protein (CRP), creatinine, Hb, RDW-standard deviation (SD), MCV, EVF and blood cell counts (erythrocytes, leukocytes, lymphocytes, neutrophils, thrombocytes). Measurements were performed at the certified central clinical laboratory at the Skåne University Hospital in Malmö, Sweden. RDW-SD was measured using a Sysmex XN-10 counter (www.sysmex.com) and was calculated as the width (femtoliters, fL) of the erythrocyte distribution curve at the relative height of 20% above the baseline. Biobanked (− 80 °C) fasting plasma samples were used for analysis of the vasopressin marker copeptin by using a KRYPTOR Compact Plus device and commercially available chemiluminescence sandwich immunoassay copeptin ProAVP kit with coated tubes (BRAHMS Copeptin proAVP KRYPTOR; ThermoFisher Scientific).

### Statistical analysis

We divided copeptin concentrations into tertiles since no cut-off values for high/low copeptin levels are established. As copeptin concentrations are known to be sex dependent, with higher concentrations among men, we used sex-specific tertiles of copeptin throughout.

Mean values of the hematopoietic markers per tertile of copeptin were compared and analyzed by an ANOVA test. Linear regression analyses were used to assess the association between increasing sex specific copeptin tertile and levels of erythrocytes, Hb, EVF, RDW-SD, MCV, leukocytes, neutrophils, lymphocytes and thrombocytes. All models were adjusted for age and sex, and in the next step additionally adjusted for BMI, current smoking, prevalent diabetes, hypertension, creatinine and leisure time physical activity as these factors have all been shown to correlate with both copeptin and the hematopoietic markers^10–24^. In addition, linear associations between SD increment in logarithmically transformed copeptin concentration and the hematopoietic markers were investigated.

To investigate whether ongoing inflammation could influence the results, a sensitivity analysis in which linear regression analyses investigating the association between increasing copeptin tertile and hematopoietic markers in strata of very low (< 3) or higher (≥ 3) CRP were performed. To exclude anemia as a possible influencing factor of the results we performed an additional sensitivity analysis, excluding anemic participants as defined above.

The level of significance was set at *p* < 0.05. All analyses were performed using the SPSS statistical software (version 28.0; SPSS, Chicago, Illinois, USA).

## Results

### Baseline characteristics

Complete data were available in 5312 individuals (mean age 57.5 years, 46.7% men), excluding individuals with incomplete data on copeptin or other covariates (n = 939, mean age 57.2 years, 47.8% men). Baseline characteristics of the included and excluded individuals are shown in Supplemental Table 1.

### Linear regression analyses

The mean values of the different hematopoietic markers according to tertiles of copeptin are shown in Table 1 and Supplemental Fig. 1a-i. Mean values of erythrocytes, RDW, EVF, Hb, leukocytes and neutrophils increased with increasing copeptin tertile, whereas there were no significant differences in mean value of MCV, thrombocytes or lymphocytes between tertiles of copeptin. In linear regression analyses, increasing copeptin tertile was significantly and positively associated with all hematopoietic markers except MCV and lymphocytes, after adjustment for age and sex. In the multivariate adjusted analyses, significant associations were found between increasing copeptin tertile and increasing erythrocytes, RDW, EVF, Hb, leukocytes, and neutrophils, respectively, after adjustment for age, sex, BMI, current smoking, prevalent diabetes, hypertension, creatinine and physical activity, whereas the association was not significant for MCV, lymphocytes and thrombocytes (Table 2). Consistent results were found in the linear regression analyses when SD increase of logarithmically transformed copeptin was used instead of copeptin tertiles, with the exception of a significant association between MCV and copeptin in the fully adjusted model (Supplemental Table 2).

**Table 1:** Hematopoietic markers in tertiles of copeptin.

Copeptin, pmol/L^a^ Copeptin, pmol/L^a^ men Copeptin, pmol/L^a^ women	Tertile 1 N = 1811 3.18 (2.66, 4.07) 4.15 (3.44, 4.77) 2.80 (2.40, 3.12)	Tertile 2 N = 1767 4.93 (4.04, 6.86) 6.97 (6.18, 7.80) 4.09 (3.76, 4.51)	Tertile 3 N = 1734 9.81 (6.87, 13.07) 12.43 (10.36, 16.31) 7.04 (5.92, 9.05)	P-value
Erythrocytes, 10^6^/μl	4.73 (0.41)	4.79 (0.43)	4.84 (0.45)	< 0.001
RDW-SD, fL	42.13 (2.88)	42.27 (3.08)	42.57 (3.28)	< 0.001
EVF, %	42.13 (3.18)	42.61 (3.25)	43.13 (3.39)	< 0.001
Hemoglobin, g/L	141.15 (11.48)	142.54 (11.85)	143.93 (12.45)	< 0.001
MCV, fL	89.29 (3.99)	89.25 (4.28)	89.28 (4.50)	0.966
Leukocytes, count/μl	5580 (1660)	5880 (1780)	6050 (2520)	< 0.001
Lymphocytes, count/μl	1870 (910)	1920 (950)	1940 (1630)	0.172
Neutrophils, count/μl	3010 (1100)	3240 (1200)	3360 (1180)	< 0.001
Thrombocytes, count/μl	251,160 (56,750)	256,760 (62,100)	255,210 (61,560)	0.016

Data expressed as mean (SD) if nothing else specified.

*RDW-SD;* red cell distribution width standard deviation, *EVF;* erythrocyte volume fraction, *MCV;* mean corpuscular volume.

^a^Expressed as median (25th, 75th percentile).

**Table 2:** Hematopoietic markers associated with increasing tertile of copeptin concentration (n = 5312).

	Beta (95% CI)	P*
Erythrocytes, 10^6^/μl		
Model 1	0.058 (0.046 – 0.070)	< 0.001
Model 2	0.039 (0.026 – 0.051)	< 0.001
RDW-SD, fL		
Model 1	0.202 (0.102 – 0.302)	< 0.001
Model 2	0.200 (0.099 – 0.302)	< 0.001
EVF, %		
Model 1	0.502 (0.413 – 0.592)	< 0.001
Model 2	0.383 (0.292 – 0.474)	< 0.001
Hemoglobin, g/L		
Model 1	1.406 (1.096 – 1.716)	< 0.001
Model 2	0.979 (0.664 – 1.293)	< 0.001
MCV, fL		
Model 1	-0.020 (-0.159 – 0.119)	0.777
Model 2	0.106 (-0.035 – 0.246)	0.140
Leukocytes, count/μl		
Model 1	235 (169 – 301)	< 0.001
Model 2	121 (56 – 186)	< 0.001
Lymphocytes, count/μl		
Model 1	36 (-3.8 – 76)	0.076
Model 2	9.0 (-31 – 49)	0.663
Neutrophils, count/μl		
Model 1	174 (136 – 212)	< 0.001
Model 2	99 (62 – 137)	< 0.001
Thrombocytes, count/μl		
Model 1	2059 (125 – 3994)	0.037
Model 2	1400 (585 – 3386)	0.167

Data expressed as unit (95% confidence interval) change in outcome variable per tertile increase in copeptin concentration.

Model 1: adjusted for age and sex.

Model 2: adjusted for age, sex, body mass index, current smoking, prevalent diabetes, hypertension, creatinine physical activity.

*RDW-SD* red cell distribution width standard deviation, *EVF* erythrocyte volume fraction, *MCV* mean corpuscular volume.

*Linear regression analysis.

### Sensitivity analysis

In the sensitivity analysis within groups of very low (< 3) or higher (≥ 3) CRP concentration (Supplemental Table 3), we found increasing copeptin tertile to be significantly associated with hematopoietic markers in both groups, except that copeptin did not remain significantly associated with leukocytes among individuals with higher CRP. In contrast, the association between copeptin and neutrophils was significant in both groups of CRP.

In the sensitivity analysis of participants classified as having anemia (n = 144), we found that the associations between increasing tertile of copeptin and increasing RDW, EVF, Hb, leukocytes and neutrophils, respectively, remained after adjustment for age, sex, BMI, current smoking, prevalent diabetes, hypertension, creatinine and physical activity. The associations remained non-significant for MCV, lymphocytes and thrombocytes (*p* > 0.05 for all).

## Discussion

In this study, the key findings are significant positive associations between copeptin and markers of both erythropoiesis as well as between copeptin and increased leukocyte and neutrophil count. The association between copeptin and the red blood cell markers (erythrocytes, RDW, Hb, EVF) could be interpreted as an indication of a more active erythropoiesis among individuals with high vasopressin levels. Our results are supported by the previous findings by Mayer et al.^4^ showing higher prevalence of permanent or intermittent anemia among individuals suffering from central diabetes insipidus. The same authors also reported expression of vasopressin receptors (AVPR1A, AVPR1B, AVPR2) in human and mouse hematopoietic stem cells, and in the mouse model, the AVPR1B receptor was found to play an important role in the regulation of erythropoiesis^4^. However, contradictory results were reported by Winzeler et al.^5^ who did not find an association between central diabetes insipidus and anemia. Their study included more detailed information about comorbidities and other possibly influencing factors. Since the latter study only evaluated anemia at one point, the two studies are not perfectly comparable and the debate concerning involvement of vasopressin in erythropoiesis is still ongoing^25^. In our study, a small part of the individuals were anemic but, as suggested by the results of a sensitivity analysis, the association between copeptin and markers of erythropoiesis was seen both among individuals classified as having anemia as well as among individuals without anemia, which in turn would indicate that the vasopressin system may be involved in stimulation of erythropoiesis independently of a low erythrocyte count. If an increase in vasopressin load, due to for example a less hydrated state, could stimulate erythropoiesis independently of the current Hb level could be an interesting subject of further research.

Copeptin was significantly associated with both leukocytes as a group, as well as neutrophil count. There was, however, no significant association with lymphocyte count. The association between leukocytes and elevated copeptin is in line with previous studies describing involvement of vasopressin in inflammation. For example, vasopressin exerts anti-inflammatory actions by involvement in the coordination of the hypothalamic–pituitary–adrenal axis activity which maintains ACTH and corticosterone levels^26^. Vasopressin is known to modulate the immune response in infectious diseases through activation on cytokines and through receptors on the leukocytes. Vasopressin induces interferon-gamma production, enhances lymphocyte response and macrophage phagocytosis^7^. Vasopressin has also been shown to stimulate the migration of monocytes and neutrophils, but when these cells were pre-incubated with vasopressin, the migratory response was decreased, illustrating the complex role of vasopressin in inflammation^2,7^. In infectious diseases, vasopressin is used in the management of septic shock but is in this situation mainly administered for its vasoconstrictive effect, since the effects on inflammation are complex^27^. There is so far no consensus on whether targeting the vasopressin system can play a positive immunomodulatory role or if vasopressin is also linked to leukocytosis in otherwise healthy individuals. Our study, demonstrating increased leukocytes and neutrophils with increased copeptin levels in healthy adults without known infection/inflammation indicates that the latter may exist. If a clear modifying effect of vasopressin stimuli on inflammation could be demonstrated, this could have interesting clinical implications.

The sensitivity analysis with stratification on CRP showed consistent results in both groups of CRP except that a significant association between copeptin and total leukocyte count was not evident among individuals with higher CRP levels (≥ 3 mg/L), whereas the association between copeptin and neutrophils remained. One may speculate that these results reflect a vasopressin stimulated leucocyte production when an inflammatory process is not present, whereas the association between copeptin and elevated neutrophils in conditions of a low-grade inflammatory process could be linked to the fact that vasopressin is a stress hormone released in response to for example low-grade infections. Thus, copeptin is not a main factor behind leukopoiesis when an inflammatory process is present. Instead, the effect of this process could be expected to override a possible subtle stimulus of copeptin on leukopoiesis.

An association between elevated copeptin and thrombocytes was seen, but the evidence of this association was, however, low after multivariate adjustment for possible confounding factors. As vasopressin is known to contribute to activation of thrombocytes by increasing serum levels of von Willebrand factor (vWF), factor VIII, and tissue plasminogen activator^2^ it could have been of interest to investigate the link between vasopressin concentration and these factors or the functional properties of thrombocytes. Neither coagulation factors nor measures of thrombocyte size or function were, however, measured in this study.

This population-based cohort study includes a large number of participants making the results generalizable to this population. All three important groups of blood cells were analyzed and evaluated in relation to copeptin, which to our knowledge has not been done before. Several aspects of erythropoiesis have also been analyzed, including both quantitative and qualitative measures, allowing a detailed investigation of its associations with the vasopressin system.

Since this study is cross-sectional it examines associations between copeptin and different measures of hematopoiesis but cannot conclude anything about causality. The possible stimulating effects of vasopressin on hematopoiesis and inflammation is proposed as ways to generate new hypotheses regarding the involvement of vasopressin in these physiological systems. Further, qualitative analysis of leukocytes and thrombocytes were unfortunately not available in the material which prevented us from analysing possible associations between vasopressin and functional measures of these blood cells.

There was a moderate number of excluded participants (n = 939). As shown by the Supplemental Table 1, these individuals were comparable to the included individuals in most parameters, including age and sex. However, diabetes mellitus, BMI, anemia, creatinine and copeptin were slightly higher/more frequent in the excluded group. This could indicate a selection bias of more healthy individuals in the included cohort.

Vasopressin is the key hormone regulating plasma osmolality. Despite large interindividual variation in water intake and water loss, plasma osmolality is kept between 285 and 295 mosm/kg due to increased vasopressin secretion when water balance is negative and reduced vasopressin secretion when water balance is positive. We have shown repeatedly that individuals with habitually low water intake express elevated copeptin concentrations which can be effectively lowered by acute or long-term water intake^28–30^. Thus, in a general population, vasopressin and copeptin could be seen as markers of an activated fluid regulation process which aims to keep body water content constant, in this way reflecting level of hydration, but should probably not be seen as markers of body water content. This is important as one may speculate in possible effects of dilution or hemoconcentration in the association between copeptin and markers of hematopoiesis. In this study, serum osmolality was unfortunately not measured. Thus, we cannot exclude the possibility that some individuals with elevated copeptin had a decreased body water content and that the association between copeptin and markers of hematopoiesis was linked to hemoconcentration. Nonetheless, the study is based on a population based sample, and none of the participants had any acute illness at the time of blood sampling. Thus, the number of individuals with slightly decreased body water content is expected to be low.

Copeptin is positively associated with indicators of increased erythropoiesis as well as increased leukocyte count in the general population. Whether the vasopressin system is mechanistically involved in erythropoiesis and leukopoiesis, and whether different vasopressin receptors mediate different effects, is an interesting area of further research. If a causal link between elevated vasopressin and hematopoiesis can be established, increased water intake constitutes a feasible intervention to lower vasopressin load which might mitigate adverse health effects of elevated erythropoiesis or leucocytosis.

### Supplementary Information

Supplementary Figures.

Supplementary Tables.

## Data Availability

The datasets generated during and/or analyzed during the current study are not publicly available due to regulations/IRB but are available from the corresponding author on reasonable request.
