# Supplementary material for: The possible role of the vasopressin system in hematopoiesis
Source: Sci Rep. 2024 Mar 1;14:5085. doi: 10.1038/s41598-024-55772-5 (PMC10907562; doi:10.1038/s41598-024-55772-5)

**Supplemental figure 1a-1i. Hematopoietic markers in tertiles of copeptin concentration.**The plots show error bars with mean values and 95% confidence intervals. The units are as follows: EVF is given in %. Erythrocytes are given in count (10^6^/μl). Hemoglobin is given in g/L. Leucocytes, neutrophils, lymphocytes, thrombocytes are given in counts (10^3^/μl). RDW and MCV are given in fL.

**Figure 1a.**


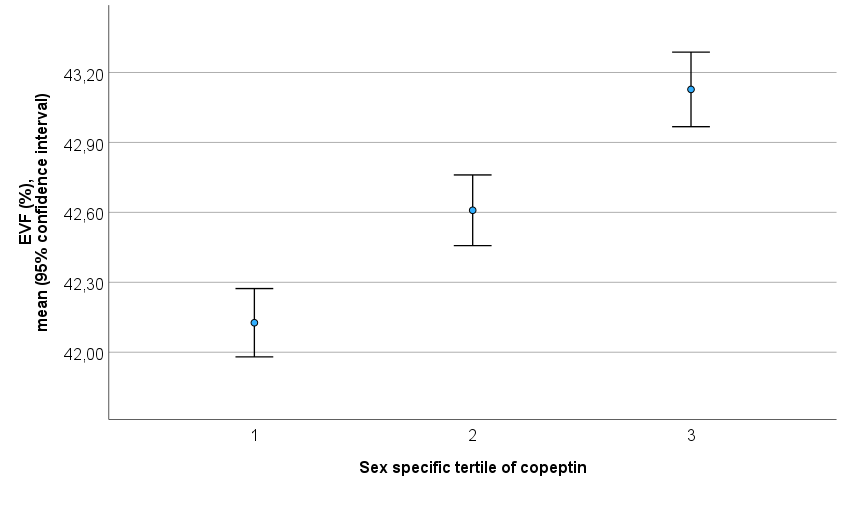


**Figure 1b.**


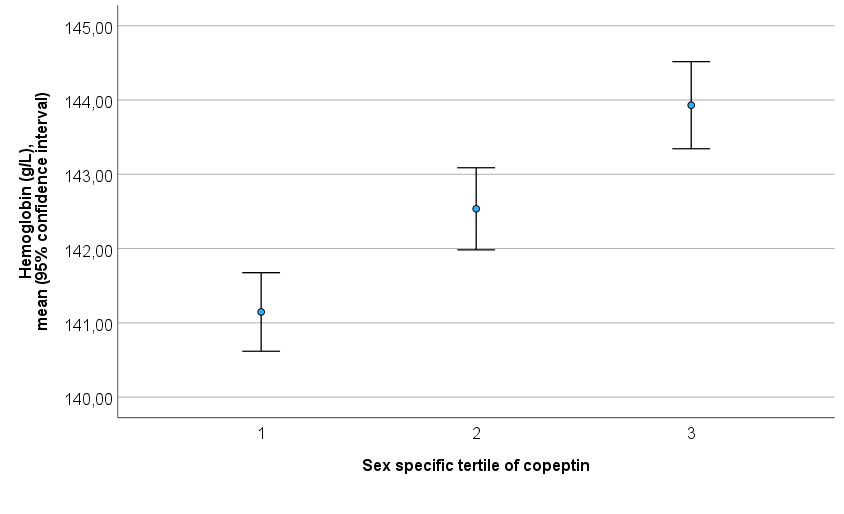


**Figure 1c.**


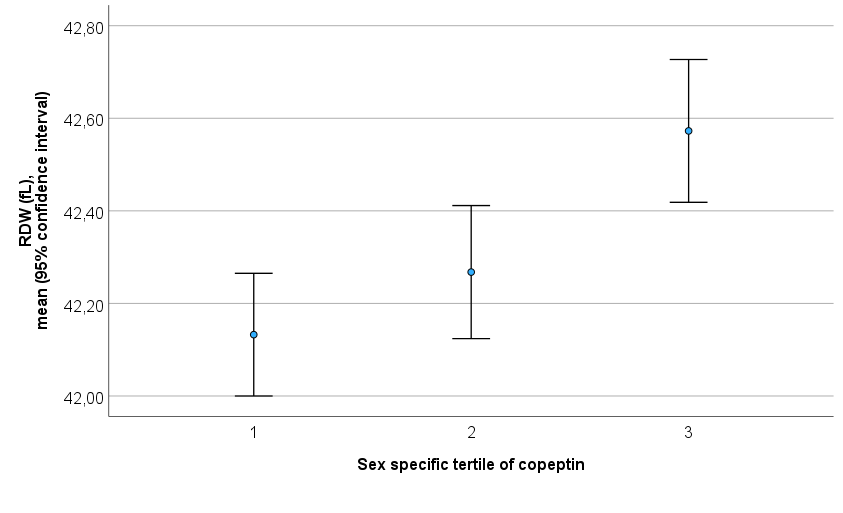


**Figure 1d.**


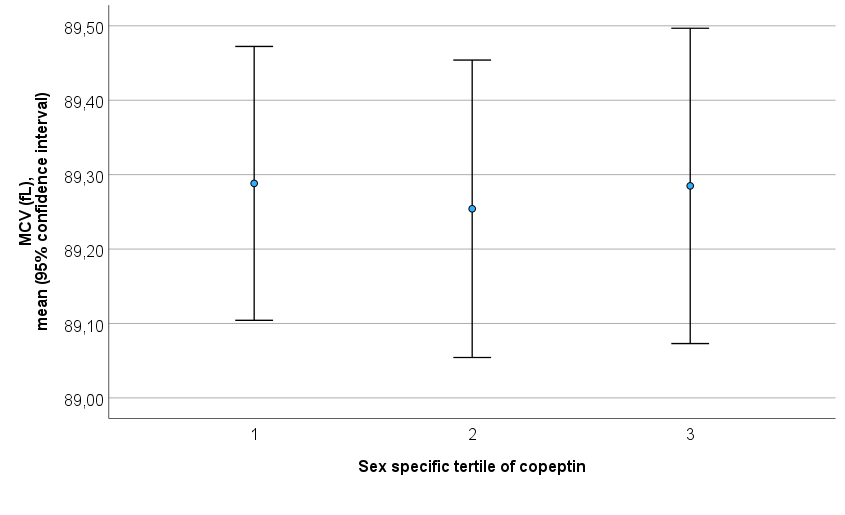


**Figure 1e.**


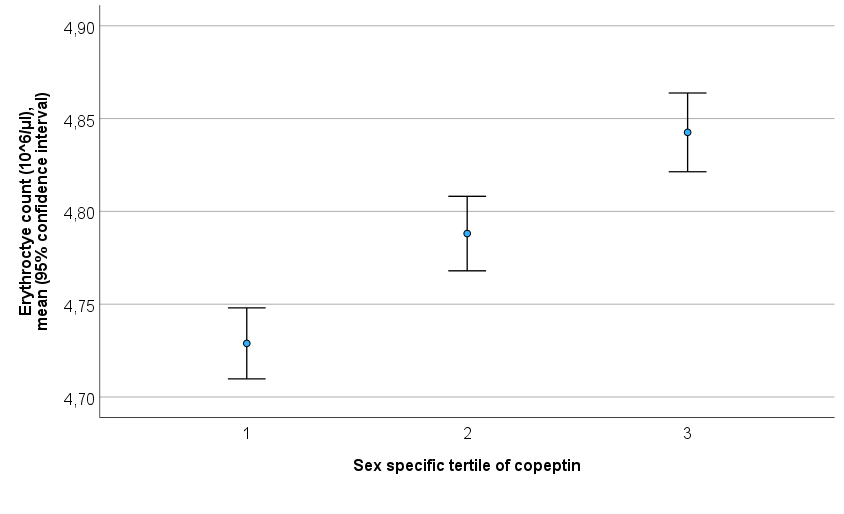


**Figure 1f.**


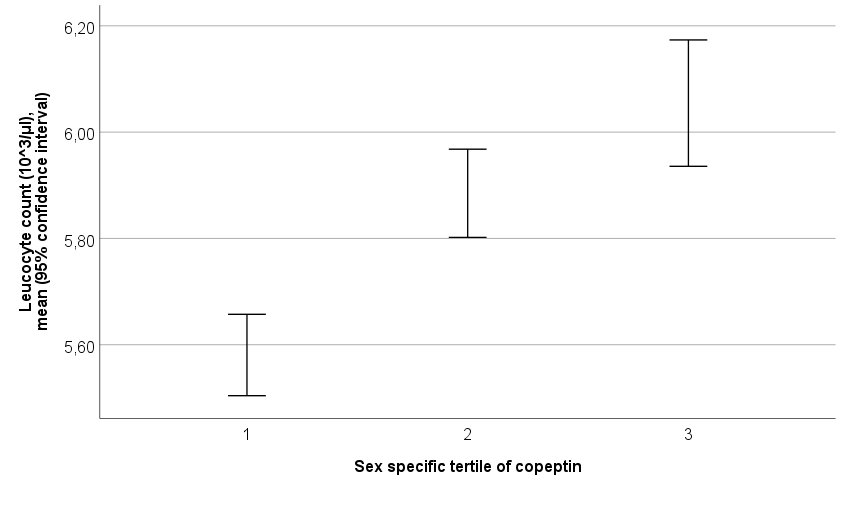


**Figure 1g.**


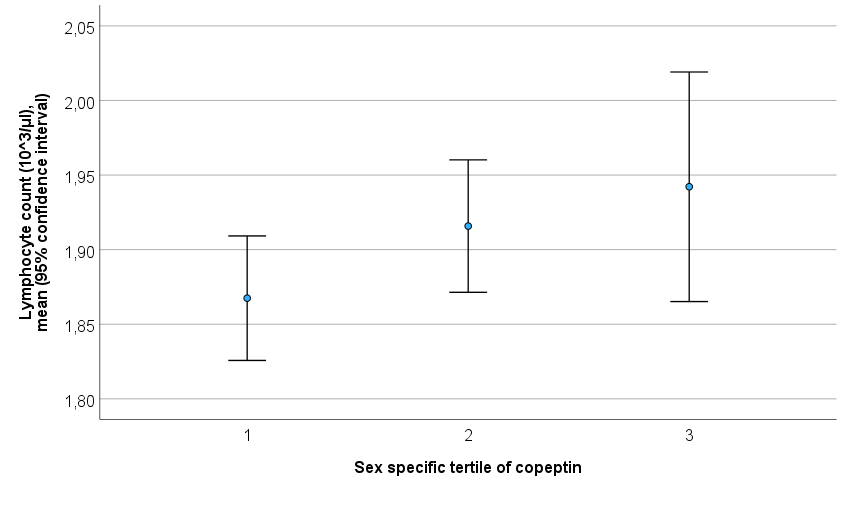


**Figure 1h.**


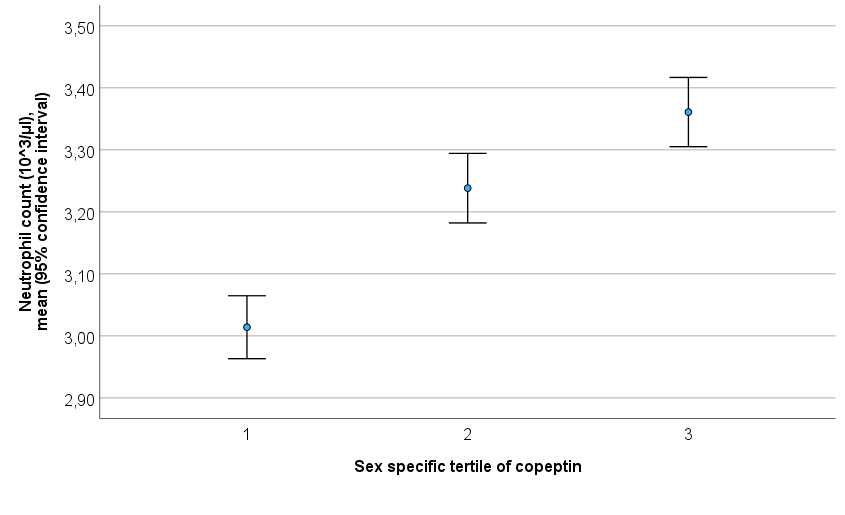


**Figure 1i.**


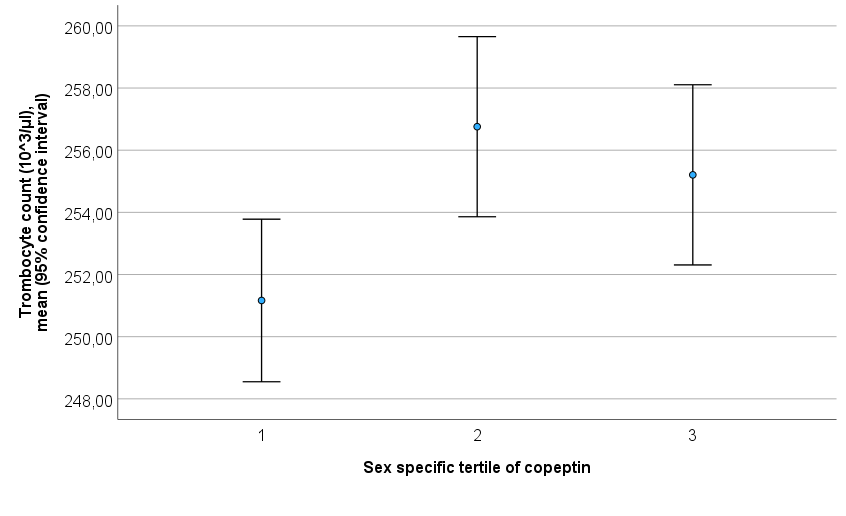

Supplement: Supplementary file 1 — Supplementary Figures. [file 41598_2024_55772_MOESM1_ESM.docx]
