# Supplementary material for: The possible role of the vasopressin system in hematopoiesis
Source: Sci Rep. 2024 Mar 1;14:5085. doi: 10.1038/s41598-024-55772-5 (PMC10907562; doi:10.1038/s41598-024-55772-5)
Supplement: Supplementary file 2 — Supplementary Tables. [file 41598_2024_55772_MOESM2_ESM.docx]

| **Supplemental table 1.** Baseline characteristics of included and excluded individuals. | | | |
| --- | --- | --- | --- |
|  | **All indviduals**  (n=6251) | **Included individuals**  (n=5312) | **Excluded individuals**  (n= 939) |
| **Age**, years  N total | 57.5 (4.3)  6251 | 57.5 (4.3)  5312 | 57.2 (4.2)  939 |
| **Men,** %  N total | 46.9  6251 | 46.7  5312 | 47.8  939 |
| **Copeptin,** pmol/L^1^  N total  **Men**  N total  **Women**  N total | 5.2 (3.6, 8.1)  5779  7.0 (4.8, 10.4)  2729  4.0 (3.13, 5.9)  3050 | 5.1 (3.6, 7.9)  5312  6.9 (4.7, 6.9)  2480  4.06 (3.1, 5.8)  2832 | 6.07 (4.0, 9.6)  467  8.1 (5.4, 12.5)  249  4.6 (3.5, 6.6)  218 |
| **Anemia**, %  N total | 3.0  6251 | 2.7  5312 | 4.4  939 |
| **Creatinine,** µmol/L  N total | 77.2 (15.0)  6223 | 77.5 (15.0)  5312 | 75.9 (15.2)  911 |
| **BMI,** kg/m2  N total | 27.3 (4.7)  6250 | 27.2 (4.5)  5312 | 29.0 (5.2)  938 |
| **Physical activity,** % in each group (1-4) ^2^  N total | 15, 49.3, 25.2, 10.4  5886 | 14.6, 49.2, 25.7, 10.4  5312 | 18.6, 50.2, 20.9, 10.3  574 |
| **Diabetes mellitus,** %  N total | 8.1  6251 | 7.7  5312 | 10.6  939 |
| **hsCRP,** mg/L^1^  N total | 1.2 (0.6-2.5)  6223 | 1.1 (0.6-2.4)  5312 | 1.4 (0.7-3.0)  911 |
| **Hypertension,** %  N total | 31.3  6251 | 31.4  5312 | 31.0  939 |
| **Erythrocytes,** 10^6^/μl  N total | 4.8 (0.4)  6194 | 4.8 (0.4)  5312 | 4.8 (0.5)  882 |
| **RDW-SD,** fL  N total | 42.3 (3.1)  6049 | 42.3 (3.1)  5312 | 42.1 (3.1)  737 |
| **MCV,** fL  N total | 89.2 (4.3)  6199 | 89.3 (4.3)  5312 | 88.8 (4.7)  887 |
| **Hemoglobin,** g/L  N total | 142.7 (12.2)  6197 | 142.5 (12.0)  5312 | 143.6 (13.3)  885 |
| **Leukocytes,** count/μl  N total | 5890 (2000)  6212 | 5840 (2200)  5312 | 6230 (2100)  900 |
| **Lymphocytes,** count/μl  N total | 1920 (1100)  6207 | 1910 (1200)  5312 | 2000 (700)  895 |
| **Neutrophils,** count/μl  N total | 3240 (1200)  6209 | 3200 (1200)  5312 | 3450 (1400)  897 |
| **Trombocytes,** count/μl  N total | 254000 (59900)  6195 | 254000 (60200)  5312 | 252000 (58100)  883 |
| Data expressed as mean (SD) if nothing else specified.  ^1^ Expressed as median (25^th^, 75^th^ percentile)  ^2^ Physical activity groups: 1= sedentary, 2= moderate exercise, 3= regular exercise, 4= regular intense exercise.  Abbreviations: BMI; body mass index, RDW-SD; red blood cell distribution width standard deviation, MCV; mean corpuscular volume. | | | |

| **Supplemental table 2**. Association between continuous increase of copeptin^1^ and hematopoietic markers (n=5312). | | | |
| --- | --- | --- | --- |
|  | **Model** | **Beta (95% CI)** | **P** |
| **Erythrocytes**, 10^6^/μl | 1 | 0.052 (0.041 - 0.063) | <0.001 |
|  | 2 | 0.036 (0.25 - 0.047) | <0.001 |
| **RDW-SD**, fL | 1 | 0.214 (0.125 - 0.304) | <0.001 |
|  | 2 | 0.223 (0.132 - 0.314) | <0.001 |
| **EVF**, % | 1 | 0.483 (0.403 - 0.563) | <0.001 |
|  | 2 | 0.392 (0.311 - 0.473) | <0.001 |
| **Hemoglobin**, g/L | 1 | 1.319 (1.041 - 1.598) | <0.001 |
|  | 2 | 0.974 (0.692 - 1.255) | <0.001 |
| **MCV**, fL | 1 | 0.037 (-0.088 - 0.162) | 0.558 |
|  | 2 | 0.153 (0.027 - 0.278) | 0.017 |
| **Leukocytes**, count/μl | 1 | 195 (135 - 254) | <0.001 |
|  | 2 | 108 (50 - 166) | 0.001 |
| **Lymphocytes**, count/μl | 1 | 24 (-12 - 59) | 0.190 |
|  | 2 | 4.5 (-32 - 41) | 0.805 |
| **Neutrophils**, count/μl | 1 | 152 (118 - 187) | <0.001 |
|  | 2 | 95 (61 - 128) | <0.001 |
| **Thrombocytes**, count/μl | 1 | 1290 (-448 - 3029) | 0.146 |
|  | 2 | 833 (-944 - 2610) | 0.358 |
| ^1^ Standard deviation increase of log-transformed copeptin concentration.  Data expressed as unit (95% confidence interval) change in outcome variable per standard deviation increase in log-transformed copeptin.  Model 1: adjusted for age and sex.  Model 2: adjusted for age, sex, body mass index, current smoking, prevalent diabetes, hypertension, creatinine, physical activity.  Abbreviations: RDW-SD; red cell distribution width standard deviation, EVF; erythrocyte volume fraction, MCV; mean corpuscular volume. | | | |

| **Supplemental table 3**. Hematopoietic markers associated with increasing tertile of copeptin concentration (n=5312) divided by CRP levels (≥ or < 3). | | | | | |
| --- | --- | --- | --- | --- | --- |
|  | **Model** | **CRP ≥ 3**  Beta (95% CI) | **P** | **CRP < 3**  Beta (95% CI) | **P** |
| **Erythrocytes**, 10^6^/μl | 1 | 0.055 (0.026-0.084) | <0.001 | 0.056 (0.042-0.069) | <0.001 |
|  | 2 | 0.036 (0.007-0.065) | 0.016 | 0.039 (0.026-0.053) | <0.001 |
| **RDW-SD**, fL | 1 | 0.397 (0.145-0.649) | 0.002 | 0.121 (0.012-0.229) | 0.030 |
|  | 2 | 0.370 (0.115-0.625) | 0.004 | 0.148 (0.039-0.257) | 0.008 |
| **EVF**, % | 1 | 0.535 (0.321-0.748) | <0.001 | 0.475 (0.376-0.574) | <0.001 |
|  | 2 | 0.431 (0.214-0.648) | <0.001 | 0.377 (0.277-0.478) | <0.001 |
| **Hemoglobin**, g/L | 1 | 1.253 (0.507-2.000) | 0.001 | 1.419 (1.076-1.762) | <0.001 |
|  | 2 | 0.923 (0.164-1.681) | 0.017 | 1.009 (0.664-1.355) | <0.001 |
| **MCV**, fL | 1 | 0.089 (-0.248-0.426) | 0.605 | -0.031 (-0.248-0.426) | 0.605 |
|  | 2 | 0.244 (-0.094-0.582) | 0.158 | 0.069 (-0.085-0.224) | 0.379 |
| **Leukocytes**, count/μl | 1 | 87 (-58 - 233) | 0.237 | 202 (129 - 275) | <0.001 |
|  | 2 | 37 (-107 - 180) | 0.617 | 123 (52 - 195) | <0.001 |
| **Lymphocytes**, count/μl | 1 | -57 (-136 - 23) | 0.162 | 48 (2 - 93) | 0.041 |
|  | 2 | -76 (-157 - 5) | 0.067 | 28 (-18 - 74) | 0.237 |
| **Neutrophils**, count/μl | 1 | 135 (31 - 239) | 0.011 | 132 (94 - 171) | <0.001 |
|  | 2 | 111 (8 - 215) | 0.035 | 83 (45 - 120) | <0.001 |
| **Thrombocytes**, count/μl | 1 | -327 (-5160 - 4507) | 0.895 | 1533 (-563 - 3629) | 0.152 |
|  | 2 | -1.2 (-4954 - 4952) | 1.000 | 1439 (-705 - 3583) | 0.188 |
| Data expressed as unit (95% confidence interval) change in outcome variable per tertile increase in copeptin concentration.  Model 1: adjusted for age and sex.  Model 2: adjusted for age, sex, body mass index, current smoking, prevalent diabetes, hypertension, creatinine, c-reactive protein, physical activity.  Abbreviations: CRP; high sensitive C-reactive protein, RDW-SD; red blood cell distribution width standard deviation, EVF; erythrocyte volume fraction, MCV; mean corpuscular volume. | | | | | |
